# Emotion and expertise: how listeners with formal music training use cues to perceive emotion

**DOI:** 10.1007/s00426-020-01467-1

**Published:** 2021-01-29

**Authors:** Aimee Battcock, Michael Schutz

**Affiliations:** 1 Department of Psychology, Neuroscience and Behaviour, McMaster University, Psychology Building (PC), Room 102, 1280 Main Street West, Hamilton, ON L8S 4K1 Canada; 2 School of the Arts, McMaster University, Hamilton, Canada

## Abstract

Although studies of musical emotion often focus on the role of the composer and performer, the communicative process is also influenced by the listener’s musical background or experience. Given the equivocal nature of evidence regarding the effects of musical training, the role of listener expertise in conveyed musical emotion remains opaque. Here we examine emotional responses of musically trained listeners across two experiments using (1) eight measure excerpts, (2) musically resolved excerpts and compare them to responses collected from untrained listeners in Battcock and Schutz (2019). In each experiment 30 participants with six or more years of music training rated perceived emotion for 48 excerpts from Bach’s *Well-Tempered Clavier *(*WTC)* using scales of valence and arousal. Models of listener ratings predict more variance in trained vs. untrained listeners across both experiments. More importantly however, we observe a shift in cue weights related to training. Using commonality analysis and Fischer Z score comparisons as well as margin of error calculations, we show that timing and mode affect untrained listeners equally, whereas mode plays a significantly stronger role than timing for trained listeners. This is not to say the emotional messages are less well recognized by untrained listeners—simply that training appears to shift the relative weight of cues used in making evaluations. These results clarify music training’s potential impact on the specific effects of cues in conveying musical emotion.

## Individual differences and musical training

The communication of musical emotion is both powerful and personal. Audiences bring their individual histories to the listening experience (Ladinig and Schellenberg 2012; Taruffi et al. 2017; Vuoskoski and Eerola 2011), responding differently to the same musical information due to differences in personality traits, experience and expertise or training. Musical training can influence the processing of musical structure (Koelsch et al. 2002; Sherwin and Sajda 2013)—including conveyed emotion. However, there is ongoing debate about whether musical training can be advantageous, with evidence both supporting (Castro and Lima 2014) and failing to demonstrate a clear training effect (Bigand et al. 2006). Here we contribute to ongoing discussion of the relationship between training and processing advantages/disadvantages by exploring a different yet complementary issue—how training affects the relative weighting of cues conveying emotion. To ensure broad relevance, we grounded this exploration in a set of well-known pieces for the piano routinely studied and performed around the world. Although this rich stimulus set poses certain analytical challenges, our application of statistical techniques borrowed from other fields allowed for a “deconstruction” of individual cue weights, affording new insight into a well-explored issue.

### Evidence for training’s effect on emotion perception in music

Some evidence suggests training shapes abilities to recognize expressed emotion. For example, in investigating the role of musicality, emotional intelligence, and emotional contagion on listeners’ perception of emotion, Akkermans et al. (2018) used recordings of three different melodies created to express seven different emotions. Participants heard all seven expressions for each melody four times over 28 trials, and rated excerpts on Likert scales representing the seven affective adjectives. Musical training emerged as the only predictor to explain for participants’ decoding accuracy. These findings support the argument that musical training affords some perceptual benefits when assessing communicated emotion.

Training benefits are also found for older musicians in contrast to younger ones (Castro and Lima 2014). In that study participants rated expressed emotion of short polyphonic excerpts on four affective 10-point intensity scales. Years of music training correlated with emotion categorization accuracy, where the middle-aged (range 40–60 years) musicians performed more accurately than non-musicians. Participants’ responses for each emotion could be predicted by various combinations of measured structural cues including tempo, mode, pitch range, dissonance, and rhythmic irregularity. Older musicians’ responses were better predicted in the model compared to non-musicians, which may be related to training advantages in recognition accuracy. Interestingly, differences emerged in the predictive strengths of some cues for negatively valenced emotions, supporting the idea that musicians use cues differently to decode emotion compared to untrained listeners.

Furthermore, changes in mode and tempo affect how listeners with musical training rate perceived valence and arousal differently than those without training (Ramos et al. 2011). Participants with at least six years of formal training on least one instrument heard excerpts consisting of different mode (seven possible Greek modes selected) and tempo (three possible tempos selected) combinations and had to select one of four emotion categories representing the excerpt. The effect of the tempo manipulations on participants’ valence ratings was greater for musical experts and the effect of mode had been modulated by participants’ musical background for both valence and arousal ratings. The authors however, found only slight differences, where both groups exhibited high responsiveness to the experimental manipulations. It is possible however, that with more years of musical training musicians would become increasingly more sensitive to these differences.

### Ambiguity in our understanding of training’s effect

Despite the literature suggesting effects of musical training on emotion perception, other evidence suggests untrained participants perform as just as well in tasks assessing accuracy and categorization within examples of music or prosody (Bigand et al. 2006; Juslin 1997; Trimmer and Cuddy 2008). As listeners gain musical knowledge from basic listening experience, it is possible music listening alone is sufficient to create ‘experienced’ listeners (Bigand and Poulin-Charronnat 2006). Although focused on induced emotions, work from Bigand et al. (2006) found emotional responses to music were only weakly influenced by expertise. In that study, participants grouped the emotions induced by excerpts of instrumental Western music similarly regardless of musical background. Interestingly, these findings occurred even though the selected stimuli included excerpts of great complexity, suggesting non-musicians are able to process subtle musical structures in Western music to discern emotion. Bigand and Poulin-Charronnat’s (2006) review highlights several studies covering a range of perceptual tasks including perceived tension and ability to anticipate musical events, which also fail to find a difference or advantage for those with musical training. However, it is unclear if there are additional, more recent studies finding a lack of training effects. This may reflect a potential publication bias to publish only significant findings (Mlinarić et al. 2017).

The effect of musical expertise remains opaque, given conflicting evidence regarding musical training’s effect (Akkermans et al. 2018; Castro and Lima 2014; Koelsch et al. 2002; Sherwin and Sajda 2013), or lack thereof (Bigand et al. 2006; Trimmer and Cuddy 2008). The current study asks participants to directly evaluate valence and arousal, unlike studies providing the possible discrete affect terms. Here we believe the dimensional measurement of emotion is a more reliable tool for rating excerpts that are less overt in their emotional message. This method is found to be more sensitive for ambiguous emotional content in music and shows higher inter-rater consistency for listener ratings of emotion (Eerola and Vuoskoski 2011).

## Present study

Our primary motivation for this study comes from interest in interpreting our recent findings regarding emotional communication in Bach’s well-known set of piano pieces *The Well-Tempered Clavier (Book 1)*. Perceptual ratings of those pieces have utility in identifying the specific contributions of cues such as timing, pitch height, and mode to emotional responses (Battcock and Schutz 2019). As part of that study, we examined differences in responses to excerpts cut to eight musical measure segments vs. “variable length” segments cut to end in locations aligned with the piece’s stated key. In other words, excerpts of varying length ensured they both started and ended in consistent modes. In an effort to maximize that study’s generalizability, we used listeners with minimal musical training. Analysis of that data raised important questions about whether more trained individuals would be more sensitive to these manipulations. This issue both complements previous research exploring trade-offs in cue weighting as a function of training, and extends inquiring to the use of complex, polyphonic stimuli frequently studied and performance around the world.

Our specific goal in these two new experiments is to compare the perceptual responses of musically trained listeners to previously collected responses of untrained listeners in an emotion perception task, building on past work using polyphonic stimuli (Castro and Lima 2014). We employ a dimensional approach to measuring emotion (Di Mauro et al. 2018; Russell 1980) in both musically trained and untrained individuals, with the goal of clarifying ongoing debate surrounding the effect of musical expertise on the decoding of emotional cues. This approach extends our previous work exploring the relationship between mode, pitch and timing (quantified as attack rate) and perceived emotion in Bach’s *Well-Tempered Clavier (WTC)—*a polyphonic 48-piece work balanced with respect to mode and widely performed and studied by musicians (Battcock and Schutz 2019). Using this stimulus set, we previously found timing information more important than mode—however that experiment used non-musicians, raising interesting questions about how training might alter the perceptual role of cues such as mode.

Research exploring the influence of musical training on perceived emotion often uses discrete models, where participants rate emotion on different affective adjective scales (Akkermans et al. 2018; Castro and Lima 2014; Gabrielsson and Juslin 1996). Although that method offers precision for the intended affective terms, it may exert priming effects for listeners. Unlike discrete models of emotion, the dimensional approach affords the ability to represent more variation in conveyed and perceived emotion (Eerola and Vuoskoski 2013). Thus, the ability to measure components of emotion on a fine-grained scale makes dimensional models better suited for detecting differences between trained and untrained listeners.

Specifically, our study involves comparing new data collected from trained musicians to previously collected data from ‘non musician’ participants with less than 1 year of musical training (Battcock and Schutz 2019). We assess these differences in two contexts (1) with excerpts from Bach’s *WTC* cut to be eight musical measures in length (2) using musically ‘resolved’ excerpts where each excerpt ends in the same nominal key as it started. The cues analyzed—attack rate (timing), mode and pitch height—represent three musical features proven to have a role in communicated musical emotion (Balkwill and Thompson 1999; Dalla Bella et al. 2001; Hevner 1935, 1937). Here, attack rate is chosen as our timing cue as it reflects both information about rhythmic structure as well as tempo. Further, we investigate the predictive weights of cues across participants with and without musical training to determine how expertise affects how listeners decode emotion in music.

## Experiment 1 (eight measure excerpts)

### Method

The following procedure and stimuli follow that of Battcock and Schutz (2019), the key aspects of which are summarized here. One exception was that these data were collected in two locations (sound attenuating booth as in the previous study, as well as a hotel meeting room). However testing equipment was consistent in both locations. The new studies also included the GoldSmith MSI following the presentation and responses to all 48 excerpts. Other procedure details followed Battcock and Schutz (2019) exactly, including the stimuli and numbers of participants.

#### Participants

To allow for the most direct comparison with our previous data, we recruited 30 participants for this experiment. Participants had $\ge$ 6 years of formal musical training from McMaster University and attendees of the Ontario Music Educators Association’s General Assembly held in Hamilton, Ontario (25 females, ages *M* = 27.36, SD = 13.69, years of training *M* = 6.73 SD = 0.45). On average, participants scored in the 71st percentile of the overall General Sophistication score and in 79th percentile on the Musical Training subscale using the Goldsmiths Musical Sophistication Index (Gold-MSI) as based on norms reported by the Müllensiefen et al. (2013). Participants’ reported trained instruments included piano, voice, flute, guitar, violin, french horn and the drum and bass, with piano reported as the principle instrument for ~ 57% of participants. Participants either received course credit, or compensation for their participation or participated as volunteers. The experiment met ethics standards according to the McMaster University Research Ethics Board.

#### Musical stimuli

Our stimuli consisted of excerpts from all 48 pieces of Bach’s *Well-Tempered Clavier (Book 1)* as recorded by Friedrich Gulda (Bach 1973). Each excerpt contained the first eight musical measures of the pieces and featured a 2-second fade out starting at the ninth measure. Excerpts lasted 7–64 s in duration (*M* = 30.2 s, SD = 13.6).

#### Cue quantification

Pitch height information is calculated with an approach initially described by Huron et al. (2010) and later used by Poon and Schutz (2015).  This involves summing duration-weighted pitch values within each measure and dividing by the sum of note durations within that measure. Attack rate calculations are based on the tempi chosen by Friedrich Gulda’s performance of the *WTC—*the recording used for this experiment*.* In addition, we re-calculated information as needed for experiment 2 (for excerpts of variable length rather than eight measures). We used attack rate rather than tempo, which is more sensitive to the combined effects of tempo and rhythmic structure. For example, Bach’s Ab Major Prelude has a higher tempo marking (108) than the Bb Major Prelude (76), yet its attack rate is considerably slower as its rhythmic structure involves fewer notes per measure (Schutz 2017). Pitch height values varied from 33.13–53.00 (*M* = 43.90, SD = 4.03) corresponding ~ F3 to ~ C#5, attack rate information for eight measure excerpts range 1.3–10.13 attacks per second (*M* = 4.91, SD = 2.18). We operationalized mode as the tonal center of the piece, as indicated by the denoted key signature of each score, coded dichotomously (0 = minor, 1 = Major).

#### Design and procedure

The experiment took place in two locations, the Ontario Music Educators Association (OMEA) general assembly held at the Sheraton in Hamilton, Ontario and McMaster University. Participants from the OMEA event filled out a consent form and completed the experiment in an isolated room. Following the consent form, participants from McMaster University completed the experiment in a sound-attenuating booth (IAC Acoustics, Winchester, US). For both testing locations, the experiment ran on PsychoPy (Peirce et al., 2019), a Python-based program on a 2014 MacBook Air (OS X 10.9.4). Participants heard stimuli at a consistent and comfortable listening level through Sennheiser HDA 200 headphones and provided responses using the MacBook’s trackpad.

Research assistants verbally instructed each participant to rate the perceived emotion after each excerpt using two scales: valence and arousal. The instructions explained valence as referring to how positive or negative the expressed emotion sounded, as rated on a scale from 1 (negative) to 7 (positive), arousal represented the energy of the emotion to be rated on a scale from 1 (low) to 100 (high). Participants had been encouraged to use to the full range of the scales and reminded to rate the emotion they heard and not the emotion they felt. Participants completed four practice trials before beginning the experiment, using recordings of the same album performed by Angela Hewitt (Bach 1998). Each participant listened to an individually randomized order of the 48 excerpts. Following the experiment, participants completed the Goldsmiths Musical Sophistication Index (Müllensiefen et al. 2014) and provided responses of familiarity to the musical stimuli (Appendix D).

### Analyses

#### Regression analysis

We assessed our cues as potential predictors for mean ratings of valence and arousal using standard linear multiple regression analysis from the R Statistical Package. The Major mode is chosen as the reference level for mode, meaning the remaining level of our categorical variable (minor) is contrasted against it in the analysis. For mean ratings of valence, all three cues, attack rate, mode and pitch height emerged as significant predictors (Table 1). For mean ratings of arousal, only attack rate emerged as a significant predictor (Table 1).

**Table 1:** Regression model for normalized attack rate, mode, pitch height on valence and arousal ratings

	Valence				Arousal			
Predictor coefficients	*B*	SE	*t*	*p*	*B*	SE	*t*	*p*
Attack rate	0.248	0.049	5.023	*p* < 0.001	0.474	0.085	5.570	*p* < 0.001
Mode	− 0.933	0.099	− 9.384	*p* < 0.001	− 0.235	0.171	− 1.372	*p* = 0.177
Pitch height	0.0102	0.0455	2.243	*p* = 0.030	0.050	0.078	0.634	*p* = 0.529
*R*^2^			0.812				0.498	
*F*			68.68				16.56	

Beta values indicate strength and direction of relationship between each predictor variable and valence and arousal ratings. Reference level for mode is Major

#### Commonality analysis

We used commonality analysis to partition the *R*^2^ of our models and clarify how much variance our predictors explain independently vs. in common with other predictors. Commonality analyses allows for a better understanding of regression models as it reveals relationships between the total, direct and indirect effects of regression predictors (Ray-Mukherjee et al. 2014). This study extends our previous use of commonality analysis by applying bootstrap methods providing confidence intervals for the estimations of cue weights. We then examined cue contributions to the bootstrapped data from the participant response using commonality analysis to decompose the *R*^2^ value into shared and unique variance of the model (Tables 2, 3).

**Table 2:** Commonality analysis for variance in listener ratings of valence (Experiment 1)

		*R*^2^_y.123_ = 0.8334	95% CIs*	% Explained
Unique to X_1_	Attack Rate	0.1233	[0.048, 0.158]	14.80%
Unique to X_2_	Mode	0.3239	[0.257, 0.427]	38.86%
Unique to X_3_	Pitch Height	0.0254	[0.011, 0.032]	3.05%
Common to X_1_ and X_2_	C (AR, Mo)	0.3350	[0.275, 0.342]	40.20%
Common to X_1_ and X_3_	C (AR, PH)	− 0.0179	[− 0.02, − 0.010]	− 2.15%
Common to X_2_ and X_3_	C (Mo, PH)	0.0684	[0.051, 0.077]	8.21%
Common to X_1_, X_2_ and X_3_	C (AR, Mo, PH)	0.0247	[− 0.026, − 0.021]	− 2.97%
	Totals	0.8334		100

*The empirical 95% CIs were computed using the percentile method on bootstrapped samples

Underline of components denote the shorthand representation used both further down the column in the Table and within figures

**Table 3:** Commonality analysis for variance in listener ratings of arousal (Experiment 1)

		*R*^2^_y.123_ = 0.5429	95% CIs*	% Explained
Unique to X_1_	Attack Rate	0.3428	[0.281, 0.371]	63.15%
Unique to X_2_	Mode	0.0191	[0.010, 0.036]	3.53%
Unique to X_3_	Pitch Height	0.0027	[0.000, 0.012]	0.49%
Common to X_1_ and X_2_	C (AR, Mo)	0.1809	[0.143, 0.213]	33.33%
Common to X_1_ and X_3_	C (AR, PH)	0.0029	[− 0.012, 0.010]	0.54%
Common to X_2_ and X_3_	C (Mo, PH)	0.0050	[0.003, 0.011]	0.93%
Common to X_1_, X_2_ and X_3_	C (AR, Mo, PH)	− 0.0106	[− 0.013, − 0.005]	− 1.96%
	Totals	0.5429		100

*The empirical 95% CIs were computed using the percentile method on bootstrapped samples

Underline of components denote the shorthand representation used both further down the column in the Table and within figures

## Results

Participants’ valence ratings (*M* = 4.20, SD = 1.57) ranged from 1 to 7 and arousal ratings (*M* = 55.98, SD = 25.04) ranged from 1 to 100. We calculated Cronbach’s alpha for listener ratings across all 48 excerpts to be *α* = 0.84 for valence ratings and α = 0.87 for arousal ratings, suggesting high internal response consistency. Ratings of valence and arousal are positively correlated (*r* = 0.39, *p* < 0.001), indicating our two dimensions did not function independently. Furthermore, there is a significant positive correlation between attack rate and mode [*r*(46) = 0.431, *p* = 0.003], demonstrating a relationship between faster attack rates and major modes. This relationship is also supported by a t-test analysis [*t*(46) = − 3.2419, *p* = 0.003].^1^ Pitch height correlated significantly with neither attack rate [*r*(46) = − 0.138, *p* = 0.350] nor modality [*r*(46) = 0.142, *p* = 0.334]. Finally, our debrief questions revealed approximately 70% of our participants reported recognizing pieces used in the experiment, with those participants reporting that they had played at least one of the pieces previously.

### Regression analysis

The three-cue predictor models accounted for 81.2% of the variance in valence ratings (adjusted *R*^2^ = 0.812), *F*(3,44) = 68.68, *p* < 0.001 in contrast to 49.8% of variance in arousal ratings (adjusted *R*^2^ = 0.498), *F*(3,44) = 16.56, *p* < 0.001. Participants’ predicted valence rating is equal to 0.549 + 0.248 (attack rate) − 0.933(mode) + 0.102 (pitch height). Valence ratings increased 0.248 for each note attack per second increase in attack rate, decreased 0.933 for the switch from major to minor mode and increased 0.102 for each increase in pitch. The predicted arousal rating is equal to 0.474 (attack rate), where arousal ratings increase 0.474 for each note attack per second increase in attack rate.

### Commonality analysis

**Fig. 1:**
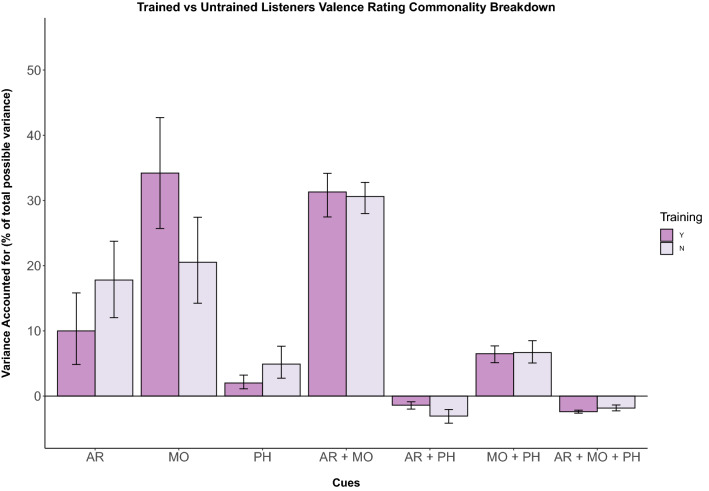
Unique and shared variance of valence ratings by musical cue. Individual bars depict cue weights calculated for each group of participants for experiment 1 (Y = musically trained, N = untrained). Error bars represent 95% confidence intervals. Attack rate uniquely explains more variance for those without musical training and modality explains a large majority of variance for those with musical training, although specific contributions vary (colour figure online)

Similar to findings from Battcock and Schutz (2019), mode accounted for the largest amount of explained variance (38.9%) in valence ratings, followed by attack rate (14.8%) and pitch height (3.1%) (Fig. 1). This indicates that mode is in fact the strongest predictor of valence ratings—even when partialling out shared variance. The combination of attack rate and mode predicted the most shared variance (40.2%) compared to shared contributions of attack rate and pitch height (− 2.15%) or mode and pitch height (8.21%) or all three cues combined (− 2.96%). The larger variance amount common to mode and attack rate is reflective of the correlation we found between these two cues.

**Fig. 2:**
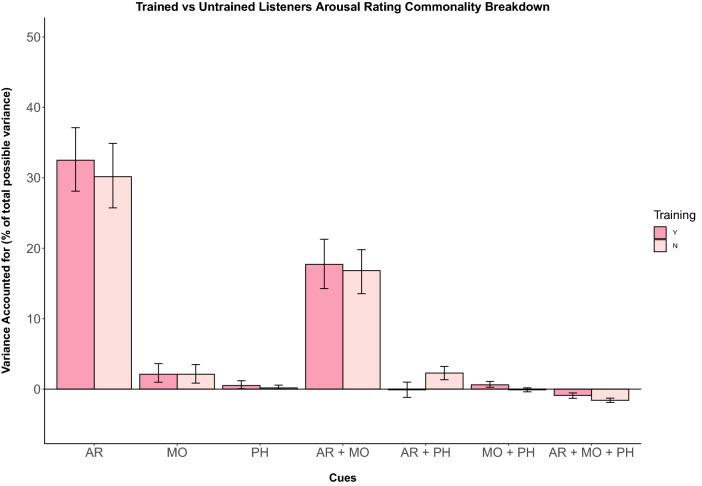
Unique and shared variance of arousal ratings by musical cue. Individual bars depict cue weights calculated for each group of participants for experiment 1 (Y = musically trained, N = untrained). Error bars represent 95% confidence intervals. Cue weights appear to explain variance similarly across participants with and without musical training (colour figure online)

For the variance of arousal ratings, attack rate is the strongest predictor, accounting for 63.2%, followed by mode (3.5%) and pitch height (0.5%) (Fig. 2). As in our model for valence ratings, the shared contribution of attack rate and mode predicted the most variance (33.3%). Contributions of other cue combinations predicted less than 1% of the model variance (Table 2).

### Comparison to untrained listener data

Comparing ratings of these musically trained participants with ratings by those without training allows for useful insight. Overall, the model for valence ratings of expert listeners accounted for more of the total variance (83.3%) than previous analyses of untrained listeners (76.2%) (Battcock and Schutz 2019). We found a similar trend for arousal, with the model for trained listeners explaining more variance (54.3%) previous analyses of untrained listeners (51.1%).

To more directly compare cue weights between the two groups of listeners, we performed Fisher’s *Z*-test to compare beta weights from trained and untrained listener models (Clogg et al. 1995; Steiger 1980). Analyses on the regression weights in models for ratings of valence show cues have equivalent weights across the two groups for attack rate (*Z* = 0.794, *p* = 0.785), mode (*Z* = − 0.989, *p* = 0.184) and pitch height (Z = 0.069, *p* = 0.755). However using this method on regression beta weights fails to address correlations between the predictors (Ray-Mukherjee et al. 2014), which play a key role in music with naturally co-varying cues (see Appendix C and Fig. 1). Therefore, we employed commonality analysis to break down the relationship between unique and shared variance explained by our predictors.

Although helpful in teasing apart the relative strength of different cues, commonality analysis does not provide a straightforward way to assess the significance of differences in cue strength. Therefore, we turned to bootstrapping to explore whether the influence of music training meaningfully increased the strength of any particular cues. Bootstrapping involves repeatedly resampling from the original data set to create multiple simulated data sets. These simulated data sets afford hypothesis testing and sample statistics in cases where these analytic solutions are not available (Mooney and Duval 1993). Our bootstrapping method used a resampling with replacement for 1000 runs simulating a sample of 30 (the same number of participants as our actual sample). Descriptive information for the bootstrapped data can be found in Appendix A.

From the generated data sets, we calculated CIs for each of the coefficients of the commonality analysis. With the bootstrapped CIs, we calculated the average margin of error (MOE) estimation for CI overlap for the coefficient representing the unique contribution of mode from our commonality analysis on the ratings of trained and untrained listeners. Using this estimation, ‘moderate’ to ‘small’ overlaps of confidence can be interpreted as equivalent to a *p* value of ≤ 0.05^2^ (Cumming 2012). In this case, moderate overlaps are calculated to be half of the average MOE of the two groups. For our data, the criterion value is 0.08 and the calculated overlap of confidence intervals is 0.02 (see Appendix C for details on the calculation), indicating that the coefficients for these two groups are likely to be significantly different from each other using an *α* level of 0.05.

## Experiment 2 (musically resolved excerpts)

Our first experiment assessed how listeners use cues of attack rate, mode and pitch to perceive emotions in musical excerpts cut to be eight musical measures in length. One limitation of using precomposed stimuli such as the *WTC* is an inability to control for modulations (or musical key changes) occurring throughout the excerpts. Therefore, we ran a second experiment as in Battcock and Schutz (2019) ensuring excerpts ended to sound musically ‘resolved’, often ending in the piece’s nominal key (e.g., the C minor excerpt for the experiment is cut at the point it returns to C minor). In many ways this offers a clearer assessment of modality’s strength, although it by definition requires excerpts with different numbers of measures. For this experiment we hypothesized (1) mode would increase in its importance for valence ratings based on ratings from those with musical training and (2) would be more important for trained compared to untrained listeners.

### Method

Experiment 2 followed the same procedure as experiment 1, but with stimuli of variable length cut to be musically ‘resolved’, often ending in the piece’s nominal key. Participants in this experiment were independent from the participants in experiment 1. As in experiment 1**,** participants included 30 individuals with $\ge$ 6 years of formal musical training from McMaster University and volunteers from the Ontario Music Educator’s Association’s General Assembly (21 females, ages *M* = 25.07, SD = 11.92, years of training *M* = 6.57 SD = 0.50). On average, participants scored in the 67th percentile on the General Sophistication scale and within the 79th percentile of the Gold-MSI Musical Training subscale. Participants’ reported trained principal instrument included piano, violin, voice, guitar, viola, saxophone and percussion, with ~ 63% of participants reporting piano as their primary instrument. Undergraduate participants received course credit, or compensation for their participation. This experiment met McMaster University Research Ethics Board ethics standards. Musical stimuli ranged from 7 to 52 s (*M* = 25.4 s, SD = 11.0).

#### Cue quantification

Pitch and timing information corresponded the quantification of each cue within the specific number of measures required to reach a ‘resolution’ back to the original mode key for each excerpt. Pitch height values varied from 33.13 to 53.13—corresponding ~ F3 to ~ C#5—(*M* = 43.87, SD = 4.15), attack rate information ranged 1.30–10.13 attacks/second (*M* = 4.87, SD = 2.22). We coded modality in the same way as in experiment 1 (0 = minor, 1 = Major).

### Results

Valence ratings (*M* = 3.94, SD = 1.58) ranged from 1 to 7 and arousal ratings (*M* = 53.78, SD = 25.33) ranged from 1 to 100. Listener ratings of valence and arousal are significantly and positively correlated *r* = 0.44, *p* < 0.001, indicating a similar lack of independence between our two dimensions as in experiment 1. The Cronbach’s alpha values for ratings across our 48 excerpts are *α* = 0.79 for valence ratings and *α* = 0.95 for arousal ratings, suggesting less consistency among listener ratings of valence than arousal (however both values fall in the acceptable range). As in experiment 1, we found a significant positive correlation between the cues of attack rate and modality [*r*(46) = 0.435, *p* < 0.001].^3^ Pitch height significantly correlated with neither attack rate [*r*(46) = − 0.165, *p* = 0.261] nor modality [*r*(46) = 0.126, *p* = 0.392]. Results from our familiarity debrief questions demonstrated 53.3% of our participants reported recognizing pieces used in experiment 2, with 53.3% of those participants reporting that they had played at least one of the pieces previously.

#### Regression analysis

As with experiment 1, we ran linear regression analyses to assess predictors for listener ratings of emotion. All three cues significantly predicted participants’ valence ratings, but only attack rate predicted arousal ratings (Table 4). The three-cue model for valence ratings accounted for 87% of variance (Adjusted *R*^2^ = 0.874), *F*(3,44) = 110, *p* < 0.001). Predicted valence ratings are equal to 2.864 + 0.167 (attack rate) − 1.923 (mode) + 0.028 (pitch height), where valence ratings increase 0.167 for each increase in note attacks per second, decrease 1.923 from the switch to minor mode and increase 0.028 for each increase in pitch height. Our arousal rating model accounted for 52% of variance (Adjusted *R*^2^ = 0.523), *F*(3,44) = 18.18, *p* < 0.001, where predicted arousal ratings are equal to 6.109 (attack rate). As such, arousal ratings increased 6.109 for each increase in note attacks per second.

**Table 4:** Regression model for normalized attack rate, mode, pitch height on valence and arousal ratings

	Valence				Arousal			
Predictor coefficients	*B*	SE	*t*	*p*	*B*	SE	*t*	*p*
Attack rate	0.106	0.022	4.892	*p* < 0.001	0.241	0.041	5.842	*p* < 0.001
Mode	− 1.220	0.095	− 12.802	*p* < 0.001	− 0.024	0.182	− 1.119	*p* = 0.269
Pitch height	0.018	0.010	1.694	*p* = 0.097	− 0.001	0.020	− 0.070	*p* = 0.944
*R*^2^			0.874				0.523	
*F*			110				18.18	

Beta values indicate strength and direction of relationship between each predictor variable and valence and arousal ratings. Reference level for mode is Major

Across the two experiments, our models for valence ratings in experiment 2 (87.4%) accounted for proportionally more total variance than in experiment 1 (81.2%). The model for arousal ratings in experiment 2 (52.3%) also accounted for proportionally similar amounts of the total variance as seen in experiment 1 (49.38%). Comparing regression weights of cues between experiment 1 and 2 illustrates that mode’s effect is significantly different (*Z* = − 1.745, *p* = 0.040). This difference in mode’s regression weight suggest mode is more predictive of valence ratings when individual pieces begin and end in the same mode. Attack rate and pitch height have equivalent regression weights in the two groups (*Z* = 0.115, *p* = 0.544 and *Z* = 0.156, *p* = 0.564, respectively), indicating no change in how listeners use these cues to make their emotion judgements.

#### Commonality analysis

**Fig. 3:**
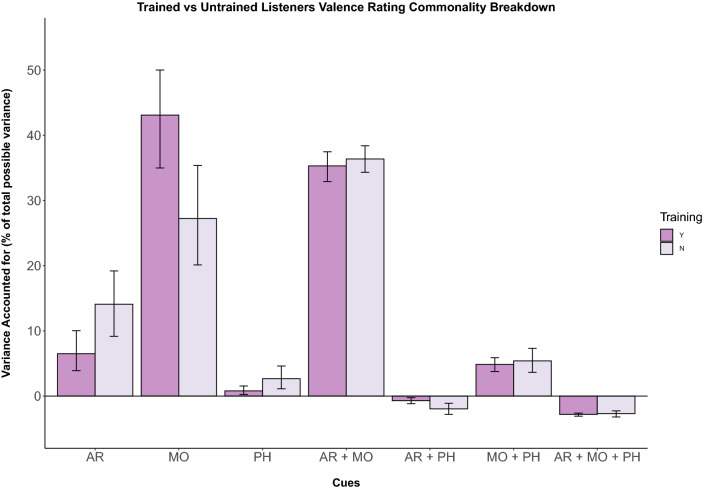
Unique and shared variance of valence ratings by musical cue. Individual bars depict cue weights calculated for each group of participants for experiment 2 (Y = musically trained, N = untrained). Error bars represent 95% confidence intervals. Attack rate uniquely explains more variance for those without musical training and modality explains a large majority of variance for those with musical training, although specific contributions vary (colour figure online)

Uniquely, mode predicted the largest amount of variance in valence responses, accounting for 49.1% (Table 5 and Fig. 3). Attack rate and pitch height contributed 8.6% and 1.6%, respectively. Attack rate and mode predicted the largest amount of shared variance (37.3%), with a small amount predicted by the shared relationship between mode and pitch height (7.7%). Values for the shared contributions between attack rate and pitch height and all three predictors remained below 0% (− 1.2% and − 3.1%, respectively).

**Table 5:** Commonality analysis for variance in listener ratings of valence (Experiment 2)

		*R*^2^_y.123_ = 0.8740	95% CIs*	% Explained
Unique to X_1_	Attack Rate	0.0759	[0.039, 0.100]	8.56%
Unique to X_2_	Mode	0.4358	[0.350, 0.500]	49.12%
Unique to X_3_	Pitch Height	0.0014	[0.002, 0.016]	1.57%
Common to X_1_ and X_2_	C (AR, Mo)	0.3310	[0.329, 0.375]	37.31%
Common to X_1_ and X_3_	C (AR, PH)	− 0.0103	[− 0.012, − 0.002]	− 1.16%
Common to X_2_ and X_3_	C (Mo, PH)	0.0681	[0.038, 0.059]	7.68%
Common to X_1_, X_2_ and X_3_	C (AR, Mo, PH)	− 0.0273	[− 0.308, − 0.026]	− 3.08%
	Totals	0.8740		100

*The empirical 95% CIs were computed using the percentile method on bootstrapped samples

Underline of components denote the shorthand representation used both further down the column in the Table and within figures

**Fig. 4:**
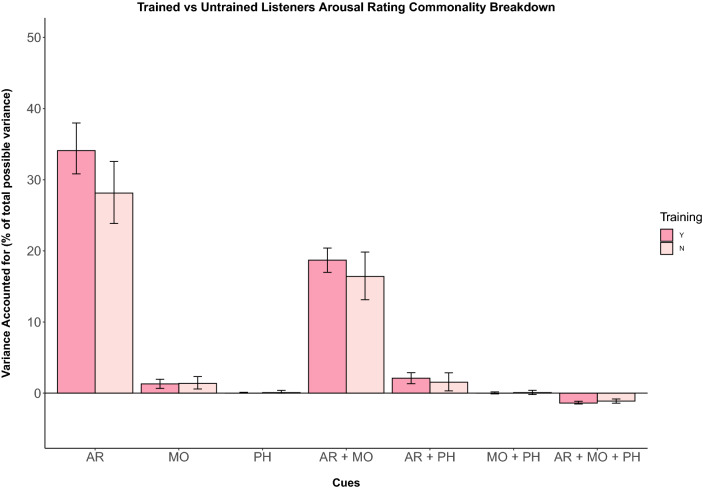
Unique and shared variance of arousal ratings by musical cue. Individual bars depict cue weights calculated for each group of participants for experiment 2 (Y = musically trained, N = untrained). Error bars represent 95% confidence intervals. Cue weights appear to explain variance similarly across participants with and without musical training (colour figure online)

As in experiment 1, the R^2^ breakdown of the model of arousal ratings (Table 6 and Fig. 4) indicates attack rate as the strongest predictor, uniquely accounting for 65.9% of the model variance. Mode and pitch height uniquely predicted only 2.6% and 0.5% of the variance from listener responses. With regards to shared contributions, only the relation between attack rate and mode predicted more than 1% of model variance (31.57%). Shared variance predicted by attack rate and pitch height accounted for 0.6%, and shared variance predicted by mode and pitch height 0.8%. The shared contribution of all three cues in the model predicted -1.86% of arousal rating variance.

**Table 6:** Commonality analysis for variance in listener ratings of arousal (Experiment 2)

		*R*^2^_y.123_ = 0.5393	95% CI*	% Explained
Unique to X_1_	Attack Rate	0.3553	[0.308, 0.380]	65.88%
Unique to X_2_	Mode	0.0137	[0.007, 0.020]	2.55%
Unique to X_3_	Pitch Height	0.0027	[0.00, 0.001]	0.49%
Common to X_1_ and X_2_	C (AR, Mo)	0.1703	[0.170, 0.204]	31.57%
Common to X_1_ and X_3_	C (AR, PH)	0.0033	[0.013, 0.029]	0.61%
Common to X_2_ and X_3_	C (Mo, PH)	0.0041	[− 0.001, 0.002]	0.77%
Common to X_1_, X_2_ and X_3_	C (AR, Mo, PH)	0.0101	[− 0.015, − 0.012]	− 1.86%
	Totals	0.5393		100

^*^The empirical 95% CIs were computed using the percentile method on 1000 bootstrapped samples

Underline of components denote the shorthand representation used both further down the column in the Table and within figures

### Comparison to non-expert data

Comparing this data with previous ratings given by untrained listeners illustrates that the model of valence ratings from trained listeners account for more of the total variance (83.3%) than untrained listeners (76.2%). We found a similar trend for arousal with more variance explained in the ratings by trained (53.9%) vs. untrained (51.1%) listeners (Fig. 4). We also calculated differences between predictors in experiment 1 and 2 for the valence ratings of untrained listeners using regression weights between experiments. This revealed mode has significantly different regression weights in experiment 1 and experiment 2 samples (*Z* = − 1.745, *p* < 0.040).

Comparing beta coefficients from our regression models for trained and untrained listeners reveal mode to have a significantly different regression weight for the model of listener responses from those with and without musical training (*Z* = − 1.854, *p* = 0.032). The cues of attack rate and pitch height have equivalent weights across the two groups (*Z* = 0.373, *p* = 0.705 and *Z* = 0.067, *p* = 0.749).

#### Comparison between experiments 1 and 2

Models of listener ratings for valence showed an increase in model fit for both trained (80–87%) and untrained listeners (76–81%) of 6–7% between experiment 1 and 2, where for both groups our three-cue model better predicted ratings in experiment 2. Regression models for the ratings of arousal demonstrated a different pattern: model fit had a slight increase between experiment 1 and 2 for trained listeners (52–55%) however decreased in fit for untrained listeners (50–46%). Results of the commonality analysis on arousal ratings indicates a difference between how our listener groups use attack rate: attack rate predicts more variance in experiment 2 compared to experiment 1 for trained listeners and predicts less for untrained listeners. Overall, the model fit appeared better for ratings from musically trained listeners, suggesting listeners with music training may use the cues more systematically than untrained listeners.

For pitch height and attack rate, the strength of their influence did not change as a result of more carefully cutting excerpts to address modulation based on the Fisher *Z* test of beta weights. However for modality we find a more nuanced outcome, with the predictive weight of mode increasing from experiment 1 to experiment 2 (*Z* = − 1.745, *p* = 0.040) for trained but not untrained (*Z* = − 1.0846, *p* = 0.140) listeners. Crucially, our commonality analyses of the bootstrapped data illustrate that mode’s unique explanatory power increases as a result of controlling more carefully for modulation. Specifically, this changes mode’s weight from 20.5 to 27.2% for untrained and from 32.4 to 43.6% for trained listeners (Tables 2, 5) when shared and unique contributions are taken into consideration (see Appendix Tables 11 and 13).

### Potential effects of familiarity for listeners with music training

**Fig. 5:**
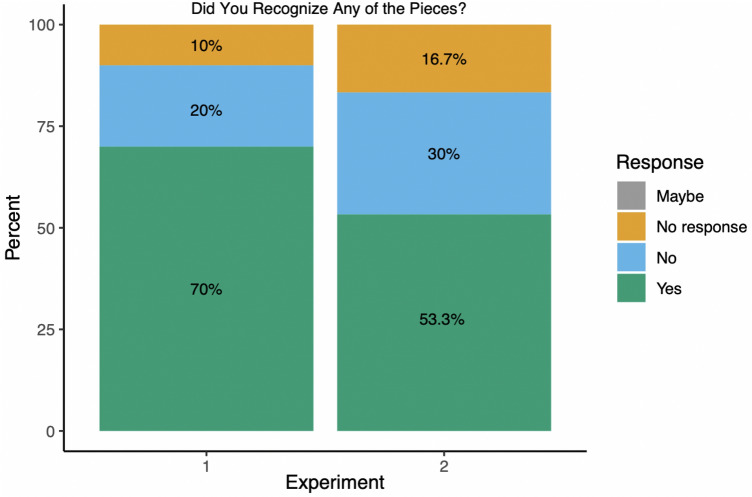
Familiarity responses from participants to “did you recognize any of the pieces” in debrief survey for experiment 1 and 2. Participants responded either yes or no, however, there were a few missed responses due to RA error. Across both experiments a large majority of participants responded they had recognized some of the excerpts presented

**Fig. 6:**
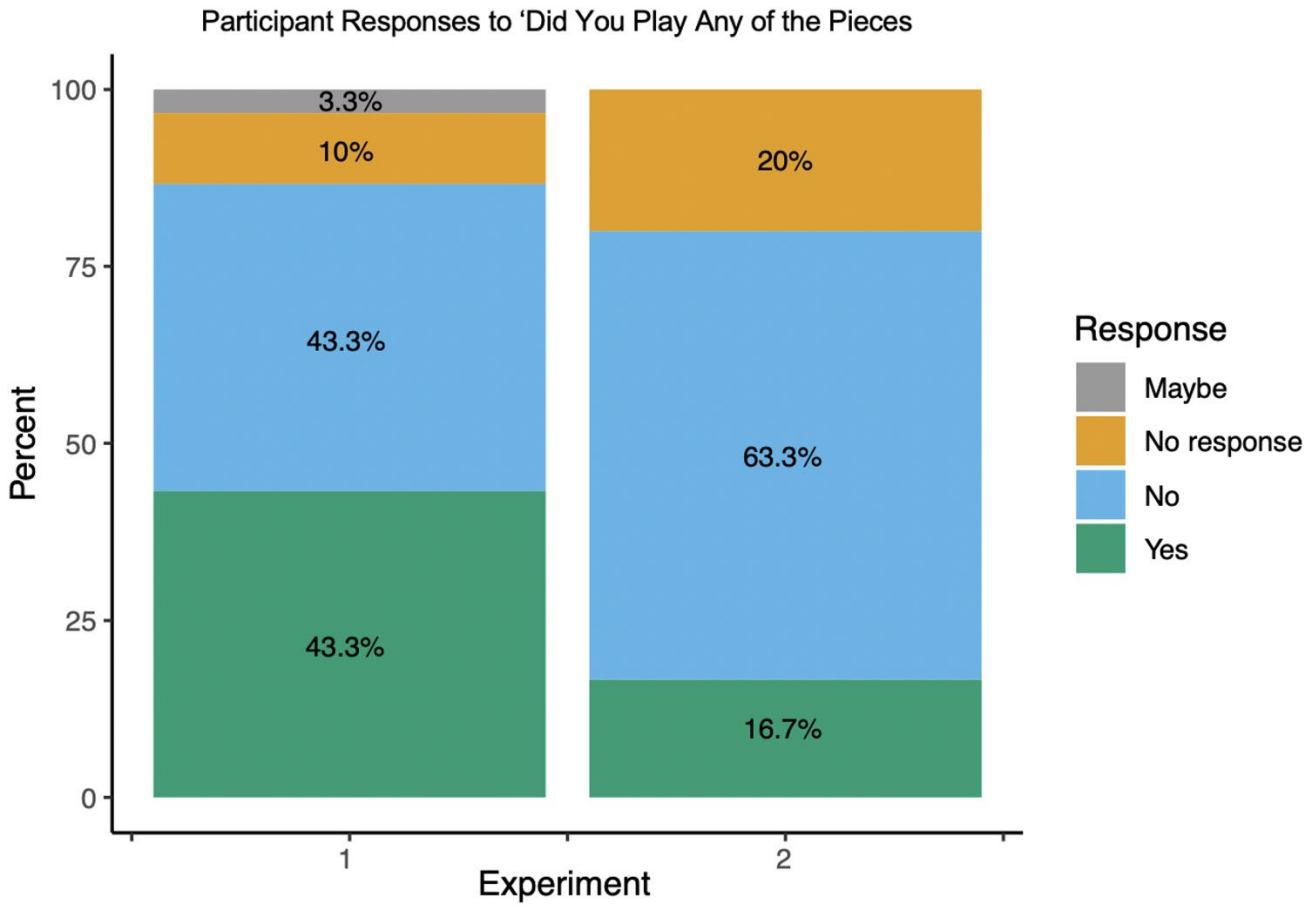
Participant responses across experiments 1 and 2 for follow-up question “have you ever played any of the pieces recognized. Participants responded either yes or no, however, there were a few missed responses due to RA error. Across both experiments the majority of responses is ‘no’, however, in experiment 1 more participants reported playing some of the pieces than in experiment 2

Following each experiment, research assistants asked participants if they had recognized any of the excerpts presented. Given the role of Western classical music in formal music training, we expected some familiarity among participants to be unavoidable. Thus, we felt it important to follow-up with general debrief questions to get a sense about whether participants recognized the excerpts presented. Based on the trained participants in our studies 70% reported recognizing excerpts in experiment 1 (Fig. 5), with some participants reporting roughly 1 to ‘a few’ excerpts appeared familiar. In experiment 2, approximately 53.33% of participants reported recognizing some of the excerpts, where some participants responded that roughly 1–3 or ‘a few’ excerpts had been familiar. Further, if the participants responded that they recognized excerpts, research assistants additionally asked if the participant had played any excerpts and could state the number played. For experiment 1, 43.3% of the total participants responded that they had played 1–5 or ‘some’ to ‘many’ (2 responses) and 16.7% responded playing some of the excerpts in experiment 2, with 1–4 or ‘some’ (1 response) (Fig. 6).

In an effort to be thorough, we ran additional regression and commonality assessments on the responses of experiment 2 as the ratio of familiar to other participants appeared more equal (53.3% ‘yes’) than within experiment 1 (70% ‘yes’). Specifically, we split our participant data based on recognition, or familiarity, and ran regression and commonality analyses on the two samples. As the motivation for this analysis came after running the study itself, the data for this exploration are both slightly imbalanced (17 participants saying ‘yes’, and 13 participants responding otherwise) and represent small sample sizes. Although these outcomes should be interpreted with caution, we include them here as they help to inform future efforts to explore the inter-relationship between training, familiarity, and emotion.

As such, the role of familiarity in this context represents both a limitation of the current study as well as an interesting direction for future research. Previous studies demonstrate that mere exposure to a stimulus can certainly affect ratings of liking (Zajonc 1968), however, it is less clear the degree to which it affects evaluations of specific dimensions of emotion. Studies of this effect in music have found evidence for familiarity increasing ratings of affect (Heingartner and Hall 1974; Peretz et al. 1998), and triggering physiological reward mechanisms (Pereira et al. 2011). At the same time, other studies show minimal contributions of familiarity to ratings most pertinent to our study—such as those of valence and arousal (van den Bosch et al. 2013). We recognize familiarity could then be playing a role in our responses, as the musically trained participants recognized some of the pieces varied between experiments. It remains unclear to what degree merely “recognizing” even one of the pieces used relates to traditional views of familiarity.

In the analyses on ratings of valence, there are differences in what predictors are significant, where pitch height is a significant predictor for participants who responded ‘yes’ to recognizing some excerpts but is not a significant predictor for the participants who did not respond ‘yes’. Overall the regression models for each group had approximately similar *R*^2^ values (*R*^2^ = 0.8671 for participants are familiar and *R*^2^ = 0.8527 for the other participants). In the commonality analysis breakdown, most notably we see mode uniquely predict more for familiar vs other participants (48% vs 29%), and attack rate predicts less (uniquely) for the familiar listeners (3.58% vs 9.09% for the other group). However, a t-test comparing mean valence ratings of each group resulted did not indicate a significant difference *t*(94) = − 0.1005, *p* = 0.9202. The t-test result suggests that there may be little variation in the mean ratings between these groups overall. However, if this can be replicated with sufficient sample sizes, the potential shift in cue weights between groups may suggest those who are familiar with the stimuli rely more on mode in perceptual judgments of emotion than attack rate, compared to those with training who are unfamiliar.

For ratings of arousal in experiment 2, regression model outputs of significant predictors are similar between participants who reported recognizing some excerpts and those who did not (see Appendix Table 17). However, *R*^2^ values for each model are *R*^2^ = 0.4568 and *R*^2^ = 0.5012 for familiar and other participant responses, respectively. As a result of similarity in predictors and small difference in R^2^ values between models, commonality analyses also demonstrated similar cue weights across unique and shared cue contributions (Appendix Table 18). In addition, we performed a t-test on mean ratings across each group which did not reach significance *t*(94) = 1.4899, *p* = 0.1398. These analyses suggest that there is little variation between the responses of the two groups.

## General discussion

The results from two new experiments involving musically trained participants (as well as comparison with two previous experiments involving untrained participants) demonstrate how training shapes the weight placed on specific musical features with respect to perceiving musical emotion. Applying new bootstrap measures to our previous work (Battcock and Schutz 2019) allows for novel comparisons between these two groups. Specifically this complements work exploring structural properties of music with listener ratings of perceived valence (Gagnon and Peretz 2003) and arousal (Schubert 2004; Vieillard et al. 2008), as we find mode more important for trained listeners in assessments of valence. Finally, these results illustrate that a model built on three cues derived from a score-based analysis can explain more variance for listeners with musical training.

Our data are consistent with the idea that trained listeners are more sensitive to particular cues than untrained listeners. Although Fisher’s *Z* score analysis on beta weights for valence ratings indicated nonsignificant differences, further analyses using commonality analysis (Table 2) and MOE calculations on bootstrapped CIs (Appendix C) revealed appreciable differences for the unique variance explained by mode (34.2% for trained listeners, 20.5% for untrained listeners). Additionally, we find mode’s greater role for trained listeners is consistent with previous developmental work showing exposure or increased experience can change the relative weight given to mode when making assessments of emotion (Dalla Bella et al. 2001). This suggests that although structural cues generally affect listeners regardless of training, the specific mix of their effects is training-dependent. This outcome is helpful in clarifying that some aspects of individual differences in the evaluation of musical emotion may be linked to different degrees of sensitivity to particular cues. In addition, these differences could in some cases stem from differential amounts of training.

Grounding this study in well-regarded music by Bach’s music offers an opportunity to explore naturally co-varying cues such as mode and timing, an issue difficult to explore when using more controlled stimuli (Schutz 2017). Although we have used commonality analysis in an exploratory manner in previous studies (Battcock and Schutz 2019), here our additional application of bootstrapping allowed us to directly assess differences in cue weights in a new way. This provides the novel insight that Bach’s decision to co-vary cues such as mode and timing results in multiple pathways for listener detection of emotion to “converge”—whether their focus is more on mode (experienced musicians) or timing (less experienced listeners). It is possible that part of the success of compositions such as the *WTC* lies in composers’ innate ability to convey messages in redundant manners. Although future research is needed to explore this issue, this outcome is one of the benefits of using the *WTC* to balance issues of musical ecological validity with experimental control.

### Musical ‘expertise’ and perception/perceptual differences

Consistent with Lima and Castro (2011), we found similar trends in the cue profiles for features predicting listener responses to auditory stimuli for both trained and untrained participants. In that study, the authors used discrete rating methods to gather emotional judgements on samples of vocal prosody and focused on regression analyses for each emotion to determine the cue profiles. Unlike their study, here we used commonality analyses in addition to regression modeling and found a difference in the strength of how mode predicted listener ratings of emotion. This novel approach illustrates that mode, a cue unique to music, predicted more variance for valence ratings for participants with musical training. Further it highlights the power of commonality analyses to tease apart the relationships between predictors and explained variance, demonstrating benefits of musical training with respect to specific cues conveying emotional information.

Previous research exploring the effect of musical training and age using monophonic, or single-lined instrumental excerpts demonstrated an influence of expertise for older participants, as years of musical trained related to recognition accuracy (Castro and Lima 2014). Their study focused on several acoustic cues such as tempo, mode and pitch range in their models of listener ratings and determined that a range of explained variance was dependent on the conveyed emotion, as well as the significant predictors of listener ratings. There, participants identified the intended emotions with high accuracy regardless of training. However models based on ratings from trained participants differed from untrained, therefore authors suggest expertise effects might be small or difficult to detect. Similarly, our results indicated differences in how the models fit for trained (80% and 52% for valence and arousal in experiment 1, 87% and 55% for experiment 2) and untrained (76% and 50% for experiment 1, 81% and 54% for experiment 2) participants, particularly for ratings of valence. This suggests differences in how these groups of listeners are using cues of attack rate, mode and pitch height to make assessments of perceived emotion.

Further, Castro and Lima (2014) found variations in how cues predicted rating variance for negative emotions such as ‘sad’ or ‘scary’, across younger and older musicians. The pattern of beta weights between trained and untrained listeners appeared similar, which the authors argue as suggesting listeners used similar inference rules in their perception of emotion. This had been determined using a multiple simultaneous regression analyses from collected intensity ratings for each of the four potential affect terms given for each excerpt. The results of our study, however, demonstrate a difference in the predictive weight of mode between trained and untrained listeners. In addition, we found the unique variance explained by mode increased more from experiment 1 to 2 for musically trained listeners than for untrained listeners, suggesting those with training were more sensitive to our resolved excerpts. As mentioned previously, differences may have emerged as a result of the stimuli used, as excerpts used in Castro and Lima (2014) represented experimentally composed excerpts, representing specific intended emotions. Our stimuli came from a precomposed set by a widely recognized composers—crafted for artistic purposes rather than for a specific research aim. It is possible that with more ambiguous stimuli, differences in cue uses may emerge when emotional signal requires more attention or consideration in the decoding process.

#### Musical training and mode

The relationship between mode and emotion is hypothesized to develop through learned associations, or acculturation from exposure and experience with Western culture music. After 5 years of age children use mode to match melodies to emotionally valenced faces (Dalla Bella et al. 2001; Gerardi and Gerken 1995; Kastner and Crowder 1990)—beforehand children predominately use timing information (Dalla Bella et al. 2001). This pattern may emerge as children use similar performance cues to decode emotion in music as is used for nonverbal aspects in speech (Juslin and Laukka 2003), consistent with findings that recognition of emotion in both music and speech develop in parallel (Vidas et al. 2018). Given that the relationship between mode and perceived emotion becomes internalized through increased knowledge and familiarity with culture-specific musical patterns, we might expect listeners with formal music training to use mode more than untrained listeners, particularly in more complex musical stimuli.

Although it has been suggested music listeners are themselves ‘experienced listeners’ (Bigand and Poulin-Charronnat 2006), those with formal music training are often instructed to use cues to express emotion and therefore may use cues differently to decode expressed emotion. Our results demonstrate mode has a stronger effect on ratings of trained listeners than those with less than 1 year of musical training. This could have occurred as a result of the complexity of the musical structure in our excerpts, leaving more ‘naïve’ listeners to use lower level cues like attack rate to understand what emotion is being transmitted, or cues commonly used to perceive emotion in vocal prosody such as timing, and loudness (Coutinho and Dibben 2013).

## Concluding thoughts

Our experiments demonstrate individuals with musical training are more affected by mode when perceiving conveyed emotion compared to untrained listeners. These results complement previous literature examining differences between behavioural and perceptual responses among musical experts and nonexperts, suggesting training can fine-tune the mechanisms used to decode musical emotions (Akkermans et al. 2018; Castro and Lima 2014; Lima and Castro 2011). In addition, our findings speak to literature exploring the role of individual differences and the effects of individual factors on emotion perception (Dibben, et al. 2018; Taruffi et al. 2017; Vuoskoski and Eerola 2011). Here we assess cue contributions, using regression analyses similar to Akkermans et al. (2018) and Eerola (2011), to model listener responses for valence and arousal. Additionaly, here we incorporate commonality analysis to examine the unique and shared predicted variance to clarify specific cue contributions. In showing differences in the influence of one particular cue (i.e., mode) over others, this work complements and extends previous research reporting conflicting results exploring training's effects on emotion perception in music. 

Previous work indicates those with musical training respond to mode-emotion associations more reliably (Heinlein 1928; Hevner 1935), however evidence also suggests training is not necessary (Dalla Bella et al. 2001). In our studies, we demonstrate the degree of mode’s effect varies as a function of training, as mode holds more weight for trained listeners than those with less than 1 year of training. Thus, individual differences in perceiving emotion can emerge between groups with and without formal music training. What requires additional investigation however is the influence of non-musical factors (SES, personality, and general cognitive ability) on emotion ratings to untangle whether our findings suggest an emotion-specific benefit or are attributed to a general cognitive advantage found in individuals who complete multiple years of formal music training. Musical competence—the ability to perceive, remember, and discriminate sequences of tones or beats—is shown to be positively associated with socioeconomic status (SES), short-term memory, general cognitive ability and the personality factor of openness (Swaminathan and Schellenberg 2018). The results from our study emerge from comparison of two groups of participants that had slight differences in average level of education and age and in testing location (sound attenuating booth at McMaster compared to hotel meeting room). Further we did not specifically collect information on other non-musical factors (SES, general cognitive ability, etc.), therefore we did not control for differences that emerge from those non-musical factors.

Additionally, the effect of familiarity should be directly explored in future efforts to unpack the inter-relationship between training, familiarity and emotion. Although our experiment captured an aspect of familiarity through debrief questions—inquiring about participants recognizing and/or playing excerpts presented—familiarity as it relates directly to our stimulus was not the focus of these studies. Familiarity is found to have some effect on increasing ratings of affect (Heingartner and Hall 1974; Peretz et al. 1998), but minimal contributions to ratings of valence and arousal (van den Bosch et al. 2013) employed in our work. Our studies looked to compare listeners with formal music training to previously collected responses of untrained listeners rating their perception of emotion to Bach’s *WTC.* The nature of using musicians with formal music training, means that familiarity to Western classical music and potentially the *WTC* specifically may be unavoidable in this context. These challenges are endemic to inquiries aiming to use both trained listeners and highly acclaimed works, as they (by their very definition) are likely to be known to a significant number of trained musicians. Therefore further research exploring the complex relationship between familiarity (i.e., mere exposure effects compared to repeated effortful playing of stimuli in training), musical training, and the communication of emotion in music will prove invaluable in clarifying our understanding of familiarity’s role in emotion perception. Exploring expertise as well as familiarity effects using additional genres of music and incorporating commonality analysis can further extend our understanding of musical training on emotion perception and more broadly, the perceptual consequences of cue use and communicated emotion. Additionally, investigating familiarity or training in non-Western cultures will help inform the relationship between cues and conveyed emotion with musical expertise in cross-cultural environments.

The influence of mode in musically expressed emotion is one that faces some debate. Although evidence demonstrates it can be effective in conveyed positive or negative affect (Hunter et al. 2008; Pallesen et al. 2005; Quinto and Thompson 2013; Webster and Weir 2005), music theorists argue its role is misunderstood (Hatten 2004). The argument is that results demonstrating mode’s influence may emerge from its relationship or pairing with other structural cues such as timing, and not an inherent binary distinction between major equals ‘happy’ and minor equals ‘sad’.

Our data help inform the debate over the emotional role of mode in at least two ways. First, they suggest mode can affect some aspects of emotion, like perceived valence, more than others, such as perceived arousal. Therefore disagreement over its role in musical emotion may stem in part from greater interest in one dimension over another. Second, these data extend traditional approaches to experimental design using systematically varied stimuli offering a high degree of independent control over individual cues such as mode and timing. Composers such as Bach essentially confounded these cues so that they co-varied—possibly to ensure robust communication of emotional messages. Consequently, disagreement over the role of mode in the communication of emotion could relate in part to different conceptions of how mode varies in passages created for scientific vs. artistic purposes.

## Appendix A

See Appendix Tables 7, 8, 9, 10.

**Table 7:** Bootstrapped commonality analysis distribution for listener ratings of valence (Experiment 1)

Bootstrapped distribution						
Cue	*N*	Coeff	SD	Min	Max	95% CI
Attack Rate	1000	0.100	0.028	0.026	0.203	[0.048, 0.158]
Mode	1000	0.342	0.044	0.206	0.471	[0.257, 0.427]
Pitch Height	1000	0.020	0.005	0.007	0.040	[0.011, 0.032]
C (AR, Mo)	1000	0.313	0.017	0.233	0.354	[0.275, 0.342]
C (AR, PH)	1000	− 0.014	0.003	− 0.023	− 0.006	[− 0.02, − 0.010]
C (Mo, PH)	1000	0.065	0.006	0.042	0.087	[0.051, 0.077]
C (AR, Mo, PH)	1000	− 0.024	0.001	− 0.028	− 0.019	[− 0.026, − 0.021]

Underline of components denote the shorthand representation used both further down the column in the Table and within figures

**Table 8:** Bootstrapped commonality analysis distribution for listener ratings of arousal (Experiment 1)

Bootstrapped distribution						
Cue	*N*	Coeff	SD	Min	Max	95% CI
Attack Rate	1000	0.325	0.022	0.251	0.396	[0.281, 0.371]
Mode	1000	0.021	0.007	0.006	0.052	[0.010, 0.036]
Pitch Height	1000	0.005	0.003	0.000	0.019	[0.000, 0.012]
C (AR, Mo)	1000	0.177	0.018	0.124	0.236	[0.143, 0.213]
C (AR, PH)	1000	− 0.001	0.005	− 0.019	0.015	[− 0.012, 0.010]
C (Mo, PH)	1000	0.006	0.002	0.001	0.015	[0.003, 0.011]
C (AR, Mo, PH)	1000	− 0.009	0.002	− 0.015	− 0.003	[− 0.013, − 0.005]

Underline of components denote the shorthand representation used both further down the column in the Table and within figures

**Table 9:** Bootstrapped commonality analysis distribution for listener ratings of valence (Experiment 2)

Bootstrapped distribution						
Cue	*N*	Coeff	SD	Min	Max	95% CI
Attack Rate	1000	0.065	0.016	0.022	0.135	[0.039, 0.100]
Mode	1000	0.431	0.038	0.273	0.551	[0.350, 0.500]
Pitch Height	1000	0.008	0.003	0.001	0.021	[0.002, 0.016]
C (AR, Mo)	1000	0.353	0.012	0.307	0.385	[0.329, 0.375]
C (AR, PH)	1000	− 0.007	0.002	− 0.014	0.000	[− 0.012, − 0.002]
C (Mo, PH)	1000	0.0486	0.005	0.029	0.066	[0.038, 0.059]
C (AR, Mo, PH)	1000	− 0.028	0.001	− 0.033	− 0.024	[− 0.308, − 0.026]

Underline of components denote the shorthand representation used both further down the column in the Table and within figures

**Table 10:** Bootstrapped commonality analysis distribution for listener ratings of arousal (Experiment 2)

Bootstrapped distribution						
Cue	*N*	Coeff	SD	Min	Max	95% CI
Attack Rate	1000	0.341	0.018	0.292	0.415	[0.308, 0.380]
Mode	1000	0.013	0.003	0.005	0.024	[0.007, 0.020]
Pitch Height	1000	0.000	0.000	0.000	0.003	[0.00, 0.001]
C (AR, Mo)	1000	0.187	0.009	0.154	0.215	[0.170, 0.204]
C (AR, PH)	1000	0.021	0.004	0.009	0.033	[0.013, 0.029]
C (Mo, PH)	1000	0.000	0.001	− 0.002	0.003	[− 0.001, 0.002]
C (AR, Mo, PH)	1000	− 0.014	0.001	− 0.016	− 0.010	[− 0.015, − 0.012]

Underline of components denote the shorthand representation used both further down the column in the Table and within figures

## Appendix B

See Appendix Tables 11, 12, 13, 14.

**Table 11:** Bootstrapped commonality analysis distribution for untrained listener ratings of valence (Experiment 1)

Bootstrapped distribution						
Cue	*N*	Coeff	SD	Min	Max	95% CI
Attack Rate	1000	0.179	0.031	0.081	0.306	[0.120, 0.238]
Mode	1000	0.205	0.034	0.093	0.332	[0.142, 0.274]
Pitch Height	1000	0.049	0.013	0.013	0.091	[0.027, 0.077]
C (AR, Mo)	1000	0.306	0.012	0.266	0.340	[0.280, 0.328]
C (AR, PH)	1000	− 0.036	0.006	− 0.049	− 0.012	[− 0.046, − 0.02,]
C (Mo, PH)	1000	0.067	0.009	0.040	0.096	[0.051, 0.085]
C (AR, Mo, PH)	1000	− 0.184	0.002	− 0.026	− 0.008	[− 0.023, − 0.014]

Underline of components denote the shorthand representation used both further down the column in the Table and within figures

**Table 12:** Bootstrapped commonality analysis distribution for untrained listener ratings of arousal (Experiment 1)

Bootstrapped distribution						
Cue	*N*	Coeff	SD	Min	Max	95% CI
Attack Rate	1000	0.302	0.024	0.219	0.387	[0.257, 0.349]
Mode	1000	0.021	0.007	0.036	0.043	[0.008, 0.035]
Pitch Height	1000	0.016	0.002	0.000	0.009	[0.000, 0.006]
C (AR, Mo)	1000	0.168	0.016	0.108	0.219	[0.135, 0.198]
C (AR, PH)	1000	0.023	0.005	0.067	0.038	[0.032, 0.010]
C (Mo, PH)	1000	− 0.001	0.001	− 0.005	0.004	[0.003, 0.011]
C (AR, Mo, PH)	1000	− 0.016	0.002	− 0.021	− 0.011	[− 0.013, − 0.005]

Underline of components denote the shorthand representation used both further down the column in the Table and within figures

**Table 13:** Bootstrapped commonality analysis distribution for untrained listener ratings of valence (Experiment 2)

Bootstrapped distribution						
Cue	*N*	Coeff	SD	Min	Max	95% CI
Attack Rate	1000	0.141	0.026	0.065	0.214	[0.092, 0.192]
Mode	1000	0.272	0.040	0.158	0.405	[0.201, 0.354]
Pitch Height	1000	0.027	0.009	0.006	0.056	[0.011, 0.046]
C (AR, Mo)	1000	0.364	0.010	0.322	0.399	[0.343, 0.384]
C (AR, PH)	1000	− 0.020	0.004	− 0.033	− 0.006	[− 0.028, − 0.011]
C (Mo, PH)	1000	0.054	0.009	0.029	0.082	[0.036, 0.073]
C (AR, Mo, PH)	1000	− 0.027	0.002	− 0.035	− 0.019	[− 0.032, − 0.023]

Underline of components denote the shorthand representation used both further down the column in the Table and within figures

**Table 14:** Bootstrapped commonality analysis distribution for untrained listener ratings of arousal (Experiment 2)

Bootstrapped distribution						
Cue	*N*	Coeff	SD	Min	Max	95% CI
Attack Rate	1000	0.281	0.022	0.216	0.360	[0.239, 0.326]
Mode	1000	0.014	0.005	0.002	0.031	[0.006, 0.023]
Pitch Height	1000	0.073	0.001	0.000	0.008	[0.000, 0.004]
C (AR, Mo)	1000	0.164	0.018	0.106	0.218	[0.131, 0.198]
C (AR, PH)	1000	0.015	0.006	− 0.006	0.036	[0.003, 0.029]
C (Mo, PH)	1000	0.008	0.002	− 0.004	0.006	[− 0.002, 0.004]
C (AR, Mo, PH)	1000	− 0.011	0.001	− 0.017	− 0.006	[− 0.014, − 0.008]

Underline of components denote the shorthand representation used both further down the column in the Table and within figures

## Appendix C

See Appendix Tables 15, 16.

**Table 15:** Margin of error calculation for valence ratings between trained and untrained listeners (Experiment 1)

Cue	Trained listeners			Untrained listeners			Avg MOE	Mod. Overlap	LCI_trained_–UCI_untrained_
	U_CI_	L_CI_	Length	U_CI_	L_CI_	Leng			
AR	0.158	0.048	0.110	0.238	0.128	0.117	0.114	0.057	− 0.189
MO	0.427	0.257	0.170	0.274	0.104	0.132	0.151	0.075	− 0.017
PH	0.032	0.011	0.021	0.077	0.055	0.049	0.035	0.018	− 0.065
AR + MO	0.342	0.277	0.067	0.328	0.261	0.048	0.057	0.029	− 0.053
AR + PH	− 0.008	− 0.020	0.011	− 0.020	− 0.032	0.021	0.016	0.008	0.000
MO + PH	0.077	0.051	0.026	0.085	0.059	0.034	0.030	0.015	− 0.034
AR + MO + PH	− 0.022	− 0.026	0.005	− 0.014	− 0.018	0.009	0.004	0.003	− 0.013

CIs represent the 95% confidence interval arousal the mean

*U*_*CI*_ Upper Confidence Interval, *U*_*LI*_ Lower Confidence Interval, *Length *length of the CI, *Avg MOE *Average Margin of Error, *Mod. Overlap *Calculated point of moderate overlap*. LCI*_*trained*_–*UCI*_*untrained*_ calculations represent the overlap calculation value

Underline of components denote the shorthand representation used both further down the column in the Table and within figures

**Table 16:** Margin of error calculation for valence ratings between trained and untrained listeners (Experiment 2)

Cue	Trained listeners			Untrained listeners			Avg MOE	Mod. Overlap	LCI_trained_–UCI_untrained_
	U_CI_	L_CI_	Leng	U_CI_	L_CI_	Leng			
AR	0.100	0.039	0.061	0.192	0.092	0.100	0.0807	0.040	− 0.153
MO	0.500	0.350	0.150	0.354	0.201	0.153	0.151	0.076	− 0.004
PH	0.016	0.002	0.013	0.046	0.011	0.035	0.024	0.012	− 0.044
AR + MO	0.375	0.329	0.046	0.384	0.343	0.041	0.043	0.022	− 0.055
AR + PH	− 0.002	− 0.012	0.009	− 0.011	− 0.028	0.017	0.013	0.007	− 0.001
MO + PH	0.059	0.038	0.021	0.073	0.036	0.037	0.029	0.0145	− 0.036
AR + MO + PH	− 0.026	− 0.031	0.005	− 0.023	− 0.032	0.009	0.007	0.004	− 0.008

CIs represent the 95% confidence interval arousal the mean

*U*_*CI*_ Upper Confidence Interval, *U*_*LI*_ Lower Confidence Interval, *Length *length of the CI, *Avg MOE *Average Margin of Error, *Mod. Overlap *calculated point of moderate overlap. *LCI*_*trained*_*–UCI*_*untrained*_ calculations represent the overlap calculation value

Underline of components denote the shorthand representation used both further down the column in the Table and within figures

## Appendix D

See Appendix Tables 17, 18.

**Table 17:** Commonality analysis for variance in listener ratings of valence between familiar and nonfamiliar participants (Experiment 2)

		R^2^		% Explained	
		Familiar	Not familiar	Familiar	Not familiar
		*R*^2^_y.123_ = 0.8756	*R*^2^_y.123_ = 0.8621		
Unique to X_1_	Attack Rate	0.0358	0.0909	4.09%	10.55%
Unique to X_2_	Mode	0.4826	0.3866	55.11%	44.84%
Unique to X_3_	Pitch Height	0.0148	0.0025	1.70%	0.29%
Common to X_1_ and X_2_	C (AR, Mo)	0.3181	0.3757	36.33%	43.58%
Common to X_1_ and X_3_	C (AR, PH)	− 0.0083	− 0.0022	− 0.95%	− 0.25%
Common to X_2_ and X_3_	C (Mo, PH)	0.0645	0.0343	7.37%	3.98%
Common to X_1_, X_2_ and X_3_	C (AR, Mo, PH)	− 0.0319	− 0.0258	− 3.64%	− 2.99%
	Totals	0.8756	0.8621	100	100

Underline of components denote the shorthand representation used both further down the column in the Table and within figures

**Table 18:** Commonality analysis for variance in listener ratings of arousal between familiar and nonfamiliar participants (Experiment 2)

		R^2^		% Explained	
		Familiar	Not familiar	Familiar	Not familiar
		*R*^2^_y.123_ = 0.4915	*R*^2^_y.123_ = 0.5330		
Unique to X_1_	Attack Rate	0.3127	0.3165	63.62%	59.4%
Unique to X_2_	Mode	0.0099	0.0162	2.01%	3.04%
Unique to X_3_	Pitch Height	0.0003	0.0011	0.07%	0.21%
Common to X_1_ and X_2_	C (AR, Mo)	0.1654	0.1883	33.65%	35.33%
Common to X_1_ and X_3_	C (AR, PH)	0.0125	0.0268	2.54%	5.03%
Common to X_2_ and X_3_	C (Mo, PH)	0.0041	− 0.0011	0.27%	− 0.21%
Common to X_1_, X_2_ and X_3_	C (AR, Mo, PH)	− 0.0106	0.0148	− 2.16%	− 2.78%
	Totals	0.4915	0.5330	100	100

Underline of components denote the shorthand representation used both further down the column in the Table and within figures

## Footnotes

1 Correlations previously reported in Battcock and Schutz (2019).

2 Although this method is not standard in hypothesis testing, the benefit of using confidence intervals instead of p-values has been argued for across different fields more generally (Ranstam, 2012; Rigby, 1999).

3 Correlations reported in Battcock and Schutz (2019).
